# Exploring trends and autonomy levels of adaptive business intelligence in healthcare: A systematic review

**DOI:** 10.1371/journal.pone.0302697

**Published:** 2024-05-10

**Authors:** João Lopes, Mariana Faria, Manuel Filipe Santos

**Affiliations:** ALGORITMI Research Center, University of Minho, Braga, Portugal; Pontifical Catholic University of Rio de Janeiro: Pontificia Universidade Catolica do Rio de Janeiro, BRAZIL

## Abstract

**Objective:**

In order to comprehensively understand the characteristics of Adaptive Business Intelligence (ABI) in Healthcare, this study is structured to provide insights into the common features and evolving patterns within this domain. Applying the Sheridan’s Classification as a framework, we aim to assess the degree of autonomy exhibited by various ABI components. Together, these objectives will contribute to a deeper understanding of ABI implementation and its implications within the Healthcare context.

**Methods:**

A comprehensive search of academic databases was conducted to identify relevant studies, selecting AIS e-library (AISel), Decision Support Systems Journal (DSSJ), Nature, The Lancet Digital Health (TLDH), PubMed, Expert Systems with Application (ESWA) and npj Digital Medicine as information sources. Studies from 2006 to 2022 were included based on predefined eligibility criteria. PRISMA statements were used to report this study.

**Results:**

The outcomes showed that ABI systems present distinct levels of development, autonomy and practical deployment. The high levels of autonomy were essentially associated with predictive components. However, the possibility of completely autonomous decisions by these systems is totally excluded. Lower levels of autonomy are also observed, particularly in connection with prescriptive components, granting users responsibility in the generation of decisions.

**Conclusion:**

The study presented emphasizes the vital connection between desired outcomes and the inherent autonomy of these solutions, highlighting the critical need for additional research on the consequences of ABI systems and their constituent elements. Organizations should deploy these systems in a way consistent with their objectives and values, while also being mindful of potential adverse effects. Providing valuable insights for researchers, practitioners, and policymakers aiming to comprehend the diverse levels of ABI systems implementation, it contributes to well-informed decision-making in this dynamic field.

## 1. Introduction

The Healthcare sector is currently undergoing a profound period of introspection, with a sharp focus on technological development and innovation. The harnessing of data generated daily through interactions between professionals and patients is deemed crucial for transforming clinical and administrative information into valuable knowledge, thereby providing steadfast support to professionals in their continual decision-making processes [1]. Artificial Intelligence (AI) is becoming more and more common in many aspects of our everyday life, yet it is still difficult to seamlessly integrate AI into professionals’ routine work in the Healthcare industry. Modern algorithms are very complex, requiring many ethical and behavioural factors to be considered while using them. There are widespread doubts about their efficacy and comprehensibility, requiring a significant amount of medical data in order to develop clinically appropriate algorithms. Interoperability and accessibility present ongoing difficulties. The enormous differences in the progress of these domains are highlighted by the quick expansion and broad acceptance of AI, which is not always accompanied by an equivalent comprehend of algorithms and outcomes. This discrepancy clarifies the medical community’s opposition to incorporating AI into Healthcare procedures [2, 3].

The vast amount of data, while also presenting itself as a valuable resource with enormous potential for use in new emerging technologies, is also an additional barrier, as the initial stages of data interpretation and preparation can be quite lengthy and complex. Feldman et al. (2018) [4] find that "the increasing availability of digitised health and wellness data has provided an exciting opportunity for complex problem analyses across the Healthcare domain", identifying a lack of insight into the type of data available for better future planning of these projects The vast diversity in data sources and intended applications often leaves researchers and Healthcare professionals with a dispersed and fragmented perspective. The introduction of Electronic Health Records (EHR) has made an essential contribution to eliminating the use of paper, providing a complete view of a patient’s medical record, including evidence-based decision support, quality management, and outcomes reporting, in addition to supporting other activities related to direct or indirect care, highlighting the need for a robust and sufficient information system capable of supporting professionals in decision making [5].

In a parallel context, Adaptive Business Intelligence (ABI) provides a structural connection between algorithms for continuous improvement and adaptation to the evolution of organisational needs, capable of delivering future insights that support user decision-making [6]. In this sector, an ABI system’s decision support function finds applications in providing information and knowledge to streamline daily tasks for Healthcare providers, enhancing service quality, and generating real-time information representations that contribute to new insights for doctors, patients, or hospital managers [7]. Despite an understanding of the theoretical and conceptual aspects covered in these domains, the synergy between AI and Healthcare remains incomplete at regulatory, ethical, socio-technical, conceptual, and methodological levels. These systems often grapple with the high level of complexity inherent in an area that demands a distinct class of interpretability, given the essential decisions made daily in such a crucial societal sector [8]. Several studies have found that the autonomy of these solutions is a central issue in the synergy that is desired to achieve and increasingly implement between these two areas. It is crucial to acknowledge that AI is not meant to supplant the human medical contribution; rather, it is designed to uphold essential functions such as patient interaction, conducting tests, comprehending and studying diagnostic procedures, and exploring therapeutic options. The primary goal is to seamlessly integrate AI into clinical workflows and decisions, positioning it as a supportive element. This integration necessitates well-prepared algorithms capable of meeting the required standards for acceptability and interpretability within these solutions [9–11].

Previous studies have addressed the application areas of an ABI system [12–16], explaining the individual type of algorithms and their applications in specific clinical processes. Upon meticulous analysis of articles related to these themes, it is noteworthy that none of them explores the definition of autonomy levels in ABI systems or their development process. Specifically, there is a gap in understanding the general characteristics and associated analytical components that contribute to the establishment of autonomy levels. This study aims to analyse a set of articles that address the different components of an ABI system, reinforcing its applicability in Healthcare but making a clear distinction of the different levels of autonomy that are proposed in each system to identify whether there was real integration into clinical workflows, as well as trends in the characteristics of its design. Thus, two research questions were formulated: What are the most common trend characteristics in an ABI System in Healthcare? (RQ1); What is the autonomy level of the ABI components based on the Sheridan’s Classification? (RQ2).

The structure of this article comprises five sections: The first, second, and third sections introduce the study areas. Subsequently, the methods employed for the systematic review are outlined, along with the resulting findings. Finally, the concluding sections, five and six, discuss pertinent remarks to the topic and propose avenues for future research.

## 2. Concepts

This section presents the theoretical concepts associated to this research, in particular the Adaptive Business Intelligence (ABI) systems and their components [6], and the classification system of autonomy proposed by Sheridan [17].

### 2.1. Adaptive business intelligence

Since the dawn of the computer age, Decision Support has stood out as one of the most critical areas in Information Technology (IT). In today’s dynamic and increasingly demanding world, this domain has become more vital than ever before. Managers are responsible for many complex decisions, such as "Should the company increase or decrease its workforce?", "Enter new markets?", "Develop new products?", "Invest in research and development?" [18]. The list is long, and if a few years ago, the support to make these decisions was very scarce, today, access to new technologies allows us to assist managers in these processes through one of the most important assets today: Data. At the core of any significant decision-making process lies two fundamental steps: Analyse the current data to understand what may happen in the future and make a final decision according to various hypotheses or perspectives considered. This thinking permeates the life of any human being today, both personally and professionally [19]. In the realm of technology, this fundamental process holds the basic premise of ABI, linked to a technological structure characterised by central components with application-oriented and conceptual framing. The foundation of this structure centres on the utilization of specific algorithms, aiming primarily to generate novel knowledge representations. Adaptive Business Intelligence (ABI) delves into four critical areas to the realization of this architecture: Artificial Intelligence, Predictive Analytics, Prescriptive Analytics, and Business Intelligence [6]. Indeed, AI, Predictive Analytics, and Prescriptive Analytics share common ground in their utilization of sophisticated algorithms and data analysis methodologies, yet they diverge in their primary functions and objectives:

1. AI is the field of science and engineering of making intelligent machines with the ability to perform tasks that would typically require human intelligence, such as learning and problem-solving and can be applied in a variety of contexts, including image recognition, natural language processing, and autonomous systems [20, 21].
2. Predictive analytics is a specific area of data analytics that focuses on forecasting future occurrences or results using machine learning algorithms and statistical models. Enables the identification of trends, patterns, and relationships in data, thereby informing decision-making processes and optimizing various aspects of business operations [22].
3. Prescriptive analytics combines forecast with optimization techniques to predict future outcomes and recommend actions to achieve a desired result. This analytical approach is applicable across diverse contexts, including supply chain management, Healthcare, and finance, where it can generate actionable recommendations for decision-makers [14].

It is understood that ABI is distinguished from Business Intelligence by the ability to implement intelligent algorithms in perspective oriented to provide future and adaptable data to the reality of the organisation, resulting in an evolution of traditional systems that focus only on historical data [6].

### 2.2. Sheridan’s Classification for Levels of Autonomy

Taking into account the specific characteristics of the Healthcare sector, the autonomy required by an AI-based system stands out as a critical factor for its successful implementation. Exploring the concept of autonomy has been a recurrent subject in many domains, especially computer systems. To provide a clear definition, we propose referring to it as "the extent to which a system can carry out its own processes and operations without external control" [23]. Based on this, Sheridan’s Autonomy Levels Classification is mentioned as the significant reference study around this concept, introducing a scale to evaluate the autonomy of a system, comprising ten levels paired with specific functional criteria [17]:

1. Level 1 gives full control to the user. In contrast, level 10 has the machine acting autonomously without informing the user.
2. Levels 2 to 4 introduce varying degrees of autonomy, distinguished by presenting all possible courses of action and recommending the most suitable course of action. In these levels, most decisions are made by the user, relying on the set of alternatives provided by the system.
3. Levels 5 to 9 exhibit several distinctions in how the user and the system make decisions. For instance, Level 5 executes the action it deems most recommended, subject to the user’s approval. Level 6 grants the user a designated time to veto the system’s recommended action. Level 7 involves an independent and autonomous execution by the system, with notification to the user. The subsequent levels (8 and 9) provide information to the user, with differences in the processing and freedom of the system to inform the user of its decisions.

Applying this methodology enables the classification of the interaction level between the system and the user as an initial requirement. This clarification helps delineate the extent of involvement and decision-making authority that Healthcare professionals will have in generating new information, considering the distinct hospital management processes in each case study.

## 3. Data and methods

The systematic review was conducted based on Preferred Reporting Items for Systematic Reviews and Meta-Analysis (PRISMA) statements [24], followed by a checklist and a flow diagram.

### 3.1. Information sources

Six data sources were selected related to research in information systems and the different areas of an ABI system with application in Healthcare. Firstly, a search was conducted in Scimago [25] covering the areas of study identified. The authors deem it pertinent to include four scientific areas (Multidisciplinary, Computer Science, Information Systems, and Biomedical) within the scope of this research, aiming to expose themselves to diverse perspectives on development and implementation. These perspectives may serve to complement or enrich the understanding of the subject under analysis. From there, journals with Q1 quartile with significant impact factors were analysed, and a set of repositories that had the potential to have articles more related to the objective of this study were selected. The Table 1 shows information about the selected data sources types.

**Table 1:** Information of selected data sources. Source: Scimago Journal and Country Rank via www.scimagojr.com, accessed on January 30, 2023.

Source	AISe-l	PubMed	DSSJ	Nature	TLDH	ESWA	npjDM
**Area**	Information Systems	Biomedical	Information Systems	Multidiscipline	Health	Computer Science	Computer Science and Medicine
**Quartile**	-	-	Q1	Q1	Q1	Q1	Q1
**H-Index**	-	-	161	1276	30	225	28
**Type**	Repository	Repository	Journal	Journal	Journal	Journal	Journal

### 3.2. Eligibility criteria and search strategy

To select the studies for the development of this systematic review, eligibility criteria were defined: (i) studies published in article format; (ii) open access or free text; (iii) written in English; (iii) articles that mention the development of predictive or prescriptive components (according to Adaptive Business Intelligence (ABI) architecture) in Healthcare; (iv) studies that contain data related to the autonomy and deployment of the developed systems/algorithms in clinical workflows. The selected articles must address practical cases related to ABI components, therefore, articles of the literature review type or that did not address a particular related topic were excluded.

The search strategy was performed considering an ordering from the most recent publications to the oldest ones, restricting to a range from 2006/01/01 to 2022/12/31, with search terms: “(("Predictive Analytics in Healthcare") OR ("Prescriptive Analytics in Healthcare"))”. The search criteria are based on the two main components of an ABI system [6], assigning a direct relationship between the levels of autonomy and the essential components properly present, in order to obtain a more general and comprehensive perspective on the type of studies obtained. The title, abstract and keywords were the main analysis strategy for each scientific publication. When these parameters were not sufficient, a complete reading of each article was made, in order to guarantee the eligibility of inclusion criteria.

### 3.3. Data extraction and management

The screening process was conducted by two authors (JL and MF) independently, involving the examination of titles, abstracts, and keywords of the search results publications. When the criteria were met, the full text was examined. In instances of disagreement or uncertainty, a third reviewer (MS) took a stance and contributed to the discussion. Through the review of eligible articles, data extraction was performed by two reviewers (JL and MF) and focused on four characteristics of an ABI system, specifically defined as: implemented components and algorithms, knowledge representation, system autonomy, and deployment. These four categories were deemed by the authors as the most relevant for the study’s objectives. Data extraction was conducted through textual analysis of articles and keyword searches. For better management and analysis, the extracted data were recorded in a suitable spreadsheet.

To address the research questions of this study, the autonomy level of each research article was identified. Based on the literature on ABI systems and the precise definition of each autonomy level [5, 26], the authors considered a set of criteria for assigning each level, as presented in Table 2. For a system to be classified at a particular level, it must meet at least one of the specified conditions.

**Table 2:** Choice of criterias based on Sheridan’s Classification for Levels of Autonomy and real deployment.

Sheridan’s Classification	Description	Condition 1	Condition 2	Condition 3
Level 1	Complete absence of system intelligence	There is no ability to predict or optimise a clinical or organisational process	It does not produce any structured information	There is no access to data to evaluate the situation
Level 2	Algorithms offer a set of results	There is no ability to predict or optimise a clinical or organisational process	Produces information about the performance of the implemented algorithms	There is access to data to evaluate the situation
Level 3	Algorithm offers several types of results with selection of best/worst	Ability to predict what may happen to the clinical/organizational situations	Produces information about the performance of the implemented algorithms	There is access to data to evaluate the situation
Level 4	Algorithms offers several types of results with best selection and restricts results to be considered in future decisions	Ability to predict various scenarios of what may happen to the clinical/ organisational situation and narrows down a set of best decisions	Produces information about the performance of the implemented algorithms and indicates possible future decisions	There is access to data to evaluate the situation
Level 5	The system performs an action with human approval	The system performs an action according to the predictive scenario obtained with human approval	The implementation of the algorithms produces structured information about the clinical/ organisational process with human approval	There is access to data to evaluate the situation
Level 6	The user has a certain time to condition an action of the system	The system performs an action according to the predictive scenario obtained with (or not) human approval	The implementation of the algorithms produces structured information about the clinical/ organisational process with (or not) human approval	There is access to data to evaluate the situation
Level 7	The system takes actions and automatically transmits information to the user	The system performs an action according to the predictive scenario obtained	The implementation of the algorithms produces structured information about the clinical/ organisational process	There is access to data to evaluate the situation
Level 8	The system takes actions automatically and transmits information to the user if he asks	The system performs an action according to the predictive scenario obtained	The implementation of the algorithms produces structured information about the clinical/ organisational process	There is access to data to evaluate the situation
Level 9	The system takes actions automati- cally and has the autonomy to trans- mit the information to the user or not	The system performs an action according to the predictive scenario obtained	The implementation of the algorithms produces structured information about the clinical/ organisational process	There is access to data to evaluate the situation
Level 10	The system takes all actions without considering the user for any kind of decision or information	The system performs an action according to the predictive scenario obtained	The implementation of the algorithms produces structured information about the clinical/ organisational process	There is access to data to evaluate the situation

### 3.4. Quality assessment and data synthesis

Each study reviewed had one or more elements of a possible ABI system designed with a particular goal. This systematic review’s main goals were to find patterns in the systems that were created, classify them, explain how to integrate data that already exists, and formulate and communicate information. Moreover, the purpose was to create links between the technological attributes and the assessment of whether a system meets the requirements for being classified as an ABI system.

## 4. Results

### 4.1. Study selection

**Fig 1:**
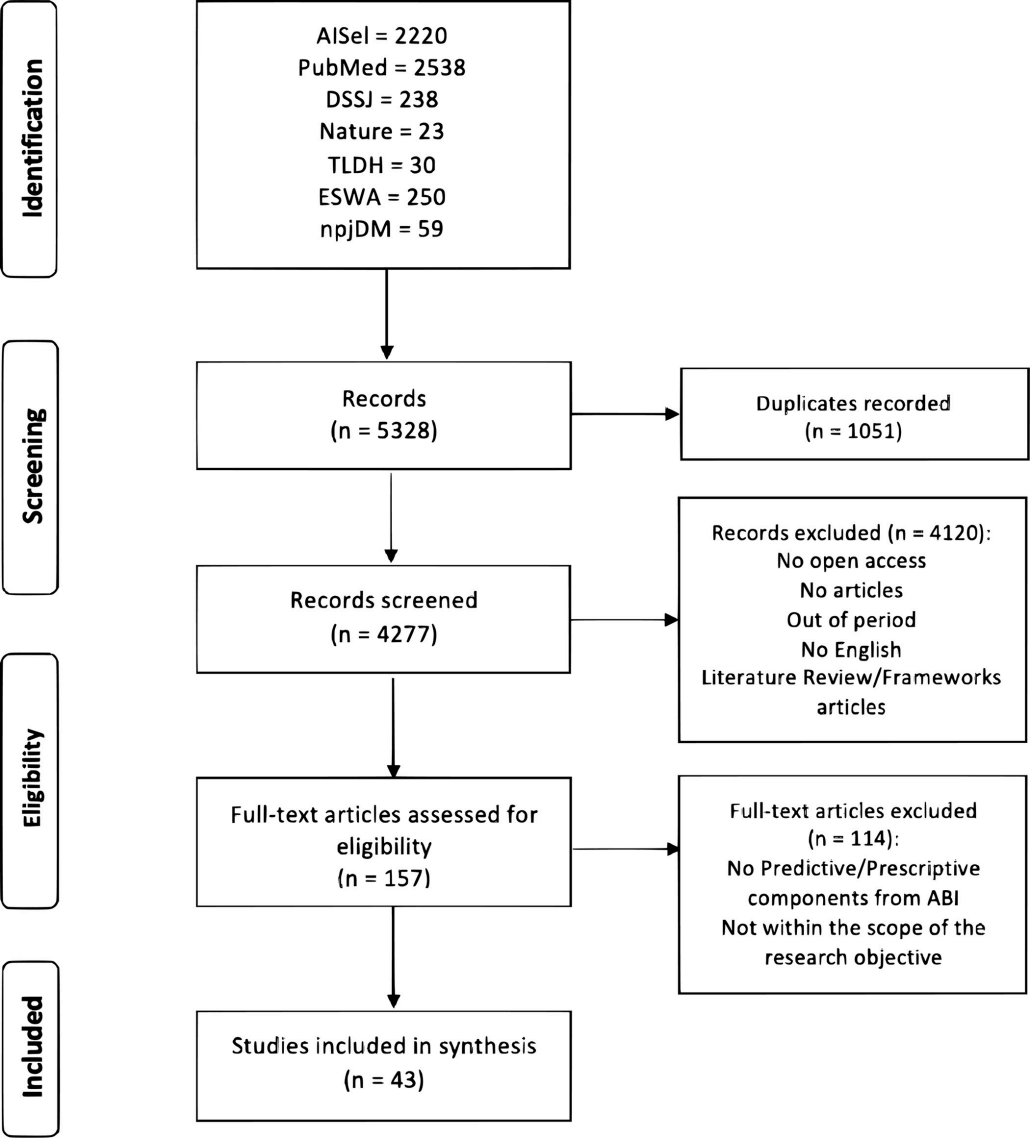
Flow diagram of study selection for systematic review.

The study was carried out following the PRISMA statement [26, 27], applying a checklist (File F1) and a flowchart represented in Fig 1. 5328 records were identified in the primary search. After removing duplicate records, 4277 records were analysed by reading the title, abstract and keywords. Subsequently, articles that did not meet the previously established selection criteria were excluded. The articles selected for the final stage totalled 157, which had to be fully read to assess their eligibility. A detailed reading of the articles was performed, leaving a final total of 43 articles that answered the RQs, which were eligible and appropriate for the systematic review.

### 4.2. Study characteristics

The studies were systematically searched across all designated data sources, with the distribution as follows: 13 studies from the Association for Information Systems e-library (30.2%), 16 studies from PubMed (37.2%), 2 studies from the Decision Support Systems Journal (6.3%), 0 studies from Nature (0.0%), 4 studies from The Lancet Digital Health (9.3%), 2 studies from Expert Systems with Applications (6.3%), and 6 studies from npj Digital Medicine (14.0%).

The period covered between studies was from 2006 to 2022, with 32,6% from 2022, prioritising the most recent studies. Studies conducted in any Healthcare setting were the most frequent (60,5%), followed by hospital setting (41,9%). The studies also reveal a broader study in Predictive Analytics (88,4%) than in Prescriptive Analytics (11,6%). Of these, 7 studies were applied to General Managers (16,3%), 23 studies to Healthcare Providers (53,5%) and 12 to both (27,9%). The overall analysis of the review articles underscores the comprehensive evaluation of different components within Adaptive Business Intelligence (ABI) systems, particularly regarding the types of algorithms implemented. Additionally, diversity is evident in the countries contributing to these studies, with the United States of America being the predominant contributor, representing 16 studies (37.2%).

### 4.3. Outcomes

The information extracted from the studies that were considered relevant for the desired purpose generated a set of outcomes, based on the characterisation of an Adaptive Business Intelligence and its main components.

1. **Study Design** is a critical aspect as it provides insights into how the research was structured. Therefore, it serves to elucidate the identified objectives of the study, as illustrated in Table 3, with three potential classifications having been established: Case Study, of which only involves a general study to the data and the effectiveness of the algorithms implemented. Prototype development, with the conceptualization of a possible product, capable of being implemented in an organisational and medical context. Cross-sectional, which is a combination of the two previous classifications. Using this structure, it was possible to identify 34 studies as Case Study (79,1%) and 9 studies as Prototype development (20,9%).
2. **Data System Integration** delineates the type of data employed in each study, discerning whether Electronic Health Records (EHR), Medical Health Records (MHR), or some specific data structure from the organization’s information system (Specific database) was utilized. Table 4 indicate that 8 studies used EHR (18,6%), 3 used MHR (7,0%), and 29 used a Specific database (67,4%). Furthermore, 2 studies incorporated EHR with a Specific database (4,7%), while only 1 study combined MHR with a Specific database (2,3%).
3. **ABI Components** is an important aspect when developing any ABI system, since a correct development planning involves understanding the usability of the predictive and/or optimization components, and how they relate to each other. To ascertain the developed components, we identified them in all studies within this systematic review, as outlined in Table 5. 38 studies used Predictive Analytics as main component for knowledge representation (88,4%) and 4 studies used Prescriptive Analytics (9,3%) and 1 study which was not possible to associate to an ABI component (2,3%).
4. **Featured Algorithms** aim to distinguish the algorithms applied, in order to identify their usability in each of the ABI components. Table 6 illustrates how we differentiated them across all the research in this systematic review to determine which ones were created. In 1 study was used Bayesian Networks (2,3%), 3 studies implemented Clustering models (7,0%), 2 studies with Decision Trees (4,7%), 6 studies implemented Deep Learning (14,0%), 1 study used Monte Carlo Simulation (2,3%), 1 study used Elastic Net (2,3%), 1 study implemented Ensemble Classifier (2,3%), 2 studies used Simulation Models (4,7%), 4 studies with Gradient Boosting (9,3%), 1 study used Extra Tree Classifier (2,3%), 5 studies used Neural Networks (11,6%), 1 study used L1R1 (2,3%), 1 study implemented Linear Regression (2,3%), 6 studies implemented Logistic Regression (14,0%), 1 study used Multivariate models (2,3%), 3 studies implemented Random Forest (7,0%), 1 study used Rule-based algorithm (2,3%), 1 study used Stacking (2,3%), 1 study used Genetic Algorithm (2,3%), 3 studies used Support Vector Machine (7,0%) and 1 study implement Time-Series (2,3%). In 3 studies, it was not possible to distinguish the implemented algorithms (7,0%).
5. **Type of System** encompasses four technological aspects, discerning the type of study presented in each article. The identified types of systems are presented in Table 7. In most of the studies (74.4%), the type of system associated is not evident, as these are only case studies where the implementation of the algorithms was not developed. In the remaining, 5 studies (11.6%) report the development and implementation of the ABI components in a software application, 4 studies (9.3%) are directed towards a web application, and only 2 studies (4.7%) focus on the development of image-based systems.

**Table 3:** Outcomes of study design.

Study	Outcome	Total	Value
[8, 24, 28–59]	Case Study	34	79.1%
[60–68]	Prototype Development	9	20.9%

**Table 4:** Outcomes of data system integration.

Study	Outcome	Total	Value
[8, 33, 36, 46, 55, 57, 58, 62]	EHR	8	18.6%
[28, 32, 37]	MHR	3	7.0%
[1, 24, 29–31, 34, 38, 40–45, 47–54, 56, 59–61, 63, 64, 66, 68]	Specific database	29	67.4%
[65, 67]	Specific database and EHR	2	4.7%
[39]	Specific database and MHR	1	2.3%

**Table 5:** Outcomes of ABI components.

Study	Outcome	Total	Value
[8, 24, 28–31, 33, 34, 36, 37, 39–44, 46–67]	Predictive Analytics	38	88.4%
[1, 38, 45, 68]	Prescriptive Analytics	4	9.3%
[32]	Not evident	1	2.3%

**Table 6:** Outcomes of featured algorithms.

Study	Outcome	Total	Value
[1]	Bayesian Networks	1	2.3%
[33, 53, 60]	Clustering	3	7.0%
[8, 56]	Decision Tree	2	4.7%
[24, 38, 43, 45, 50, 63]	Deep Learning	6	14.0%
[45]	Monte Carlo Simulation	1	2.3%
[52]	Elastic Net	1	2.3%
[68]	Ensemble Classifier	1	2.3%
[36, 39, 57, 67]	Gradient Boosting	4	9.3%
[65, 68]	Simulation Models	2	4.7%
[46]	Extra Tree Classifier	1	2.3%
[30, 37, 53, 54, 66]	Neural Networks	5	11.6%
[65]	L1R1	1	2.3%
[28]	Linear Regression	1	2.3%
[29, 31, 34, 40, 47, 51]	Logistic Regression	6	14.0%
[61]	Multivariate Models	1	2.3%
[55, 58, 64]	Random Forest	3	7.0%
[41]	Rule-based algorithm	1	2.3%
[44]	Stacking	1	2.3%
[38]	Genetic Algorithm	1	2.3%
[38, 48, 59]	Support Vector Machine	3	7.0%
[42]	Time Series	1	2.3%
[32, 49, 62]	Not evident	3	7.0%

**Table 7:** Outcomes of type of system.

Study	Outcome	Total	Value
[8, 28–34, 36–44, 46–59, 64]	Not associated	32	74.4%
[36, 45, 62, 65, 68]	Software app	5	11.6%
[24, 60, 61, 66]	Web based systems	4	9.3%
[63, 67]	Image based system	2	4.7%

**Fig 2:**
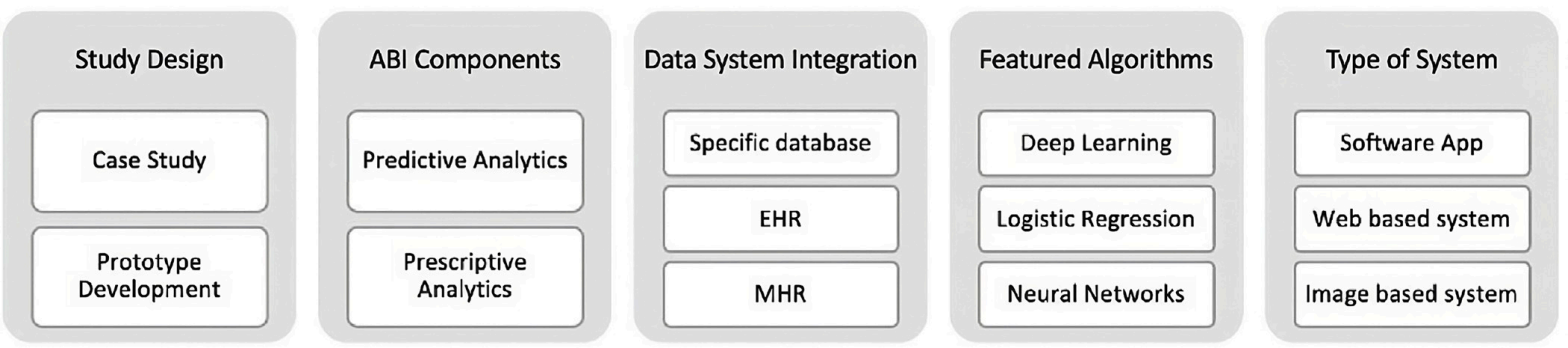
General trends obtained through the review.

The results for each analysed topic varied based on the objectives of the considered studies. Given the diversity across the studies, discernible trends in weighted topics have been identified. Fig 2 illustrates the most prominent categories. At the Study Design level, the articles classified as Case Studies stand out, where only possible algorithms were evaluated without any reference to possible implementations. There are also, but less frequently, articles with prototype development, focusing not only on algorithm evaluation but also on ABI component schematisation. Concerning Data System Integration, the studies that used specific databases are highlighted, using a varied set of data. Additionally, the studies that used EHRs and MHRs as their primary data source are also represented. Analysing the ABI Components, the predictive studies represent 79.1% of the sample. The development of prescriptive components, using predictive and optimisation models, are also present in the articles analysed, with the remaining 20.1%. In the main algorithms, the techniques of Deep Learning, Logistic Regression and Neural Networks predominate. Finally, at the level of the type of system, not forgetting the high percentage of articles where it is not evident how the algorithms could be implemented in the organisation, it is possible to mention some tendency towards systems based on software applications, as well as Web based, and Image based systems.

### 4.4 Temporal evolution

**Fig 3:**
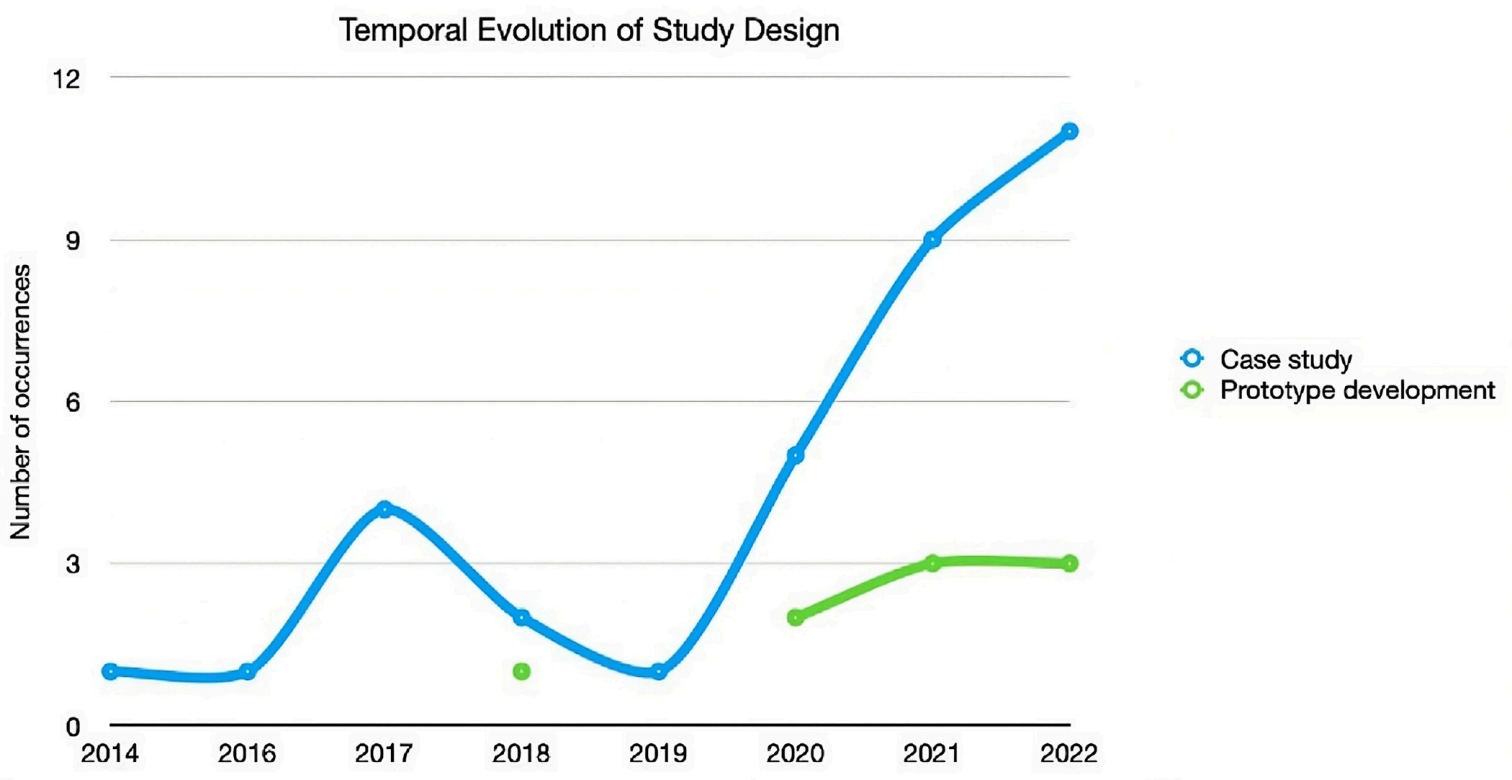
Temporal evolution of study design.

The following chapter seeks to contextualise the previous analysis topics from a temporal perspective. Fig 3 demonstrates the type of studies that have been carried out in the years considered, from which it is possible to identify the significant representation of Case Studies, i.e., the type of articles published in this area continue to address, for the most part, the performance of algorithms, with no emphasis on how these can be implemented or integrated into clinical systems.

**Fig 4:**
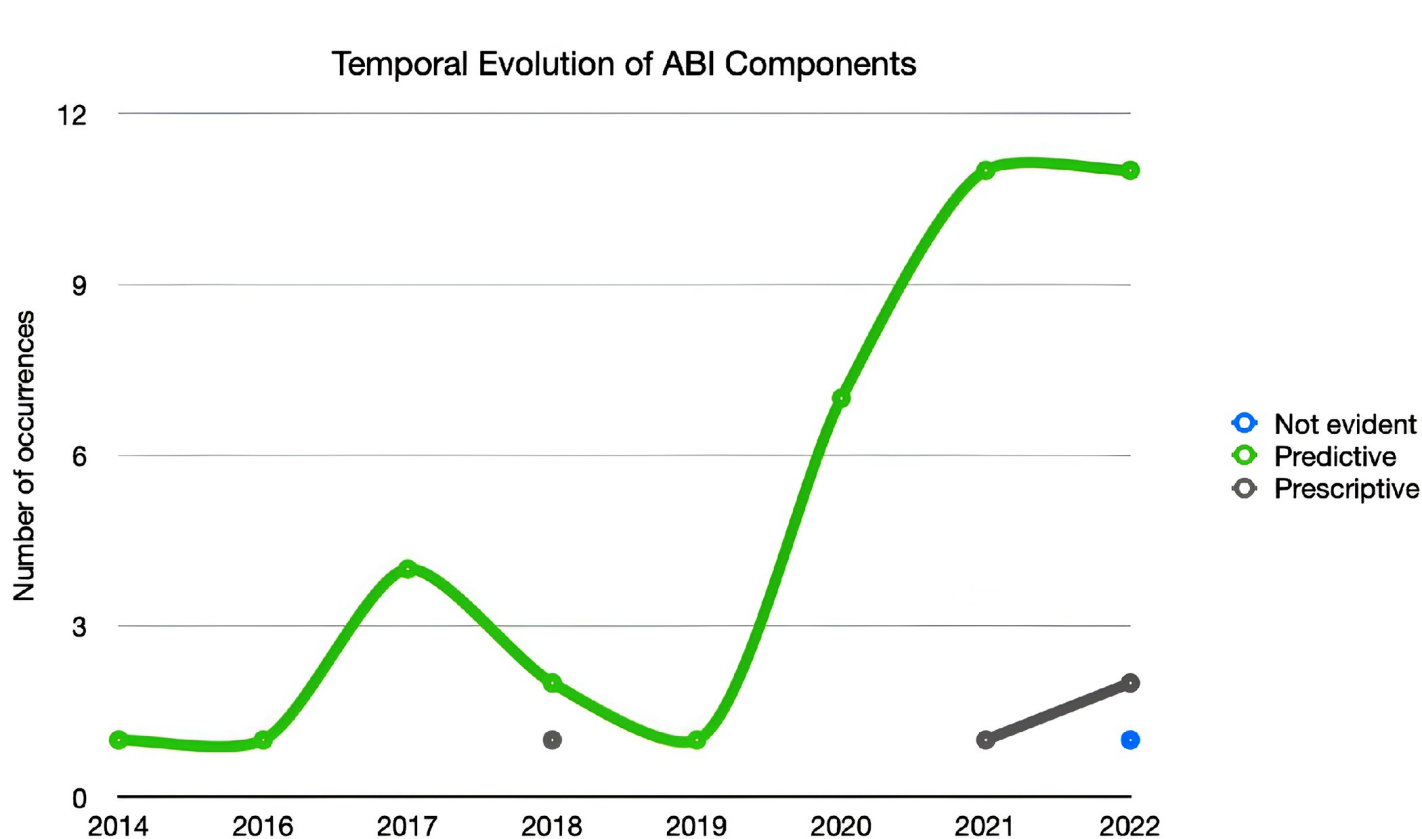
Temporal Evolution of ABI components.

It is also possible to verify a slight increase, starting in 2021, of articles referring to possible prototypes. According to Fig 4, we can see that the development of ABI components continues to be done, most prominently by Predictive Analytics. Even so, a slight increase in Prescriptive Analytics should be highlighted, which, together with the accentuated growth of the previous component, may indicate some reversal of this trend, something that will necessarily have to be monitored in the coming years.

**Fig 5:**
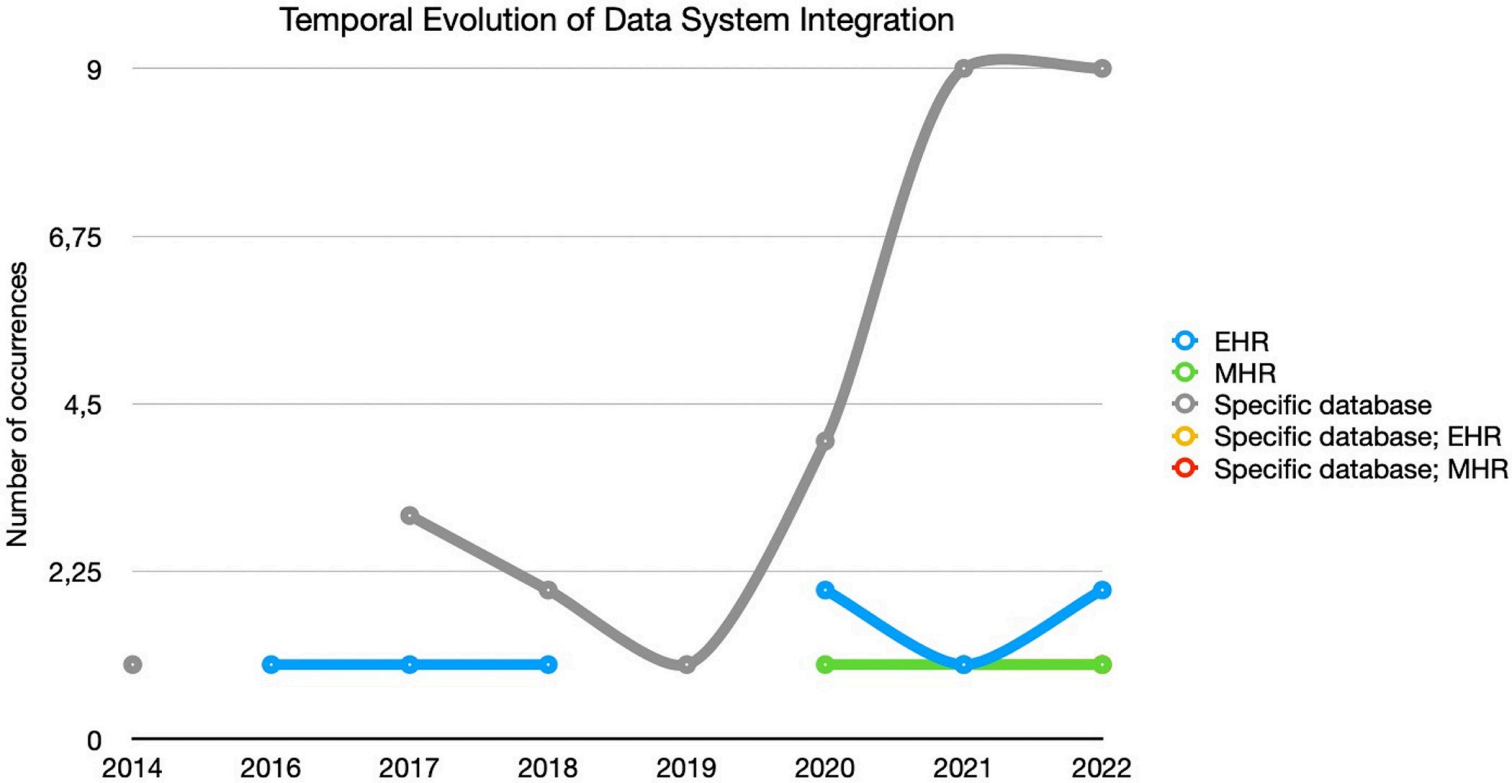
Temporal evolution of data system integration.

In Fig 5 it is possible to observe the strong representation of studies in which specific databases are used for this type of study. This indicates that the number of studies using EHR and MHR is still scarce, probably due to the strong restrictions of researchers/organisations to clinical and medical data, sustained by data protection regulation policies.

**Fig 6:**
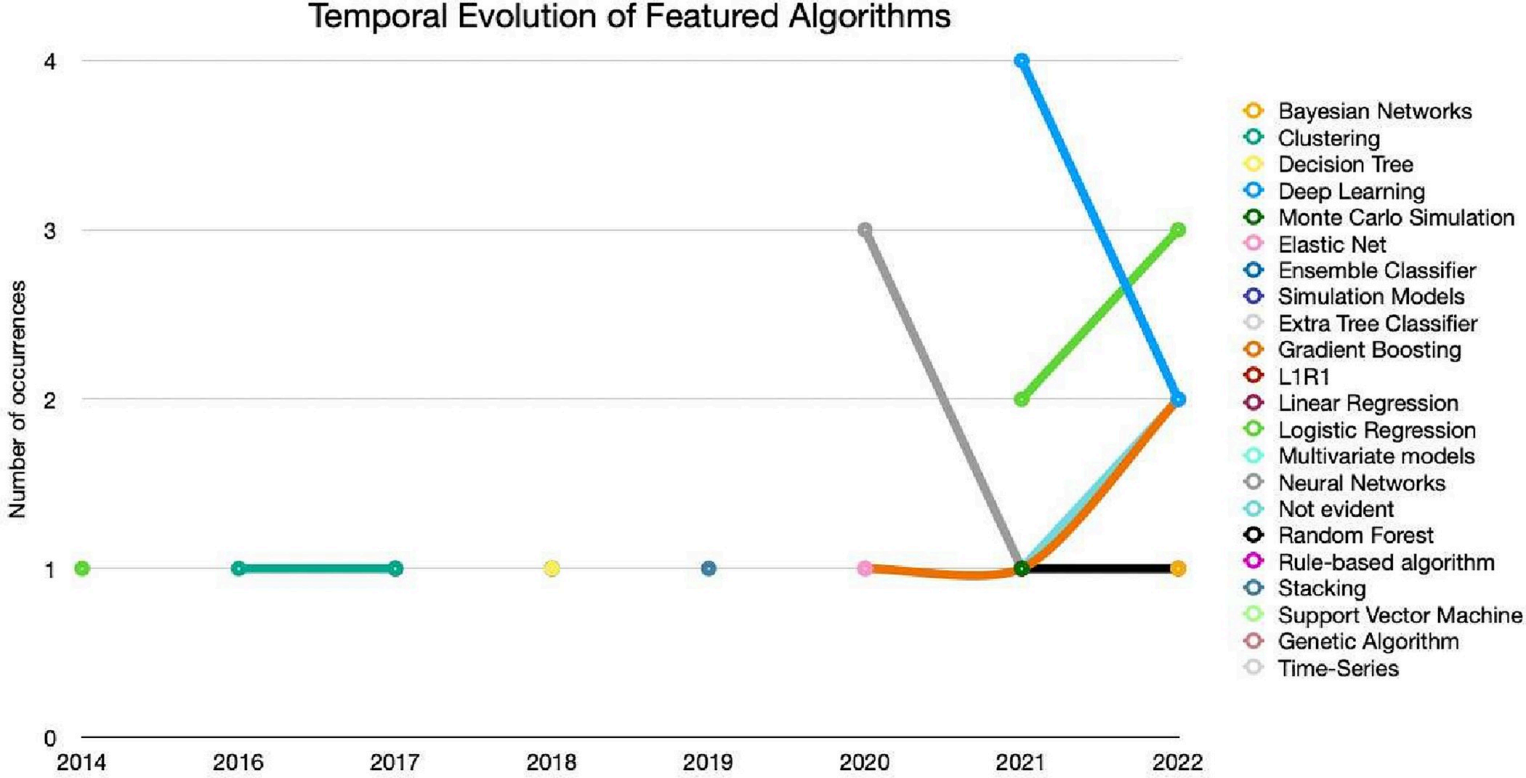
Temporal evolution of featured algorithms.

As expected, there is clear diversity in the type of algorithms implemented, as seen in Fig 6. Still, the decrease in algorithms such as Deep Learning and Neural Networks should be highlighted, in contrast to the increase in the use of Logistic Regression and Gradient Boosting.

**Fig 7:**
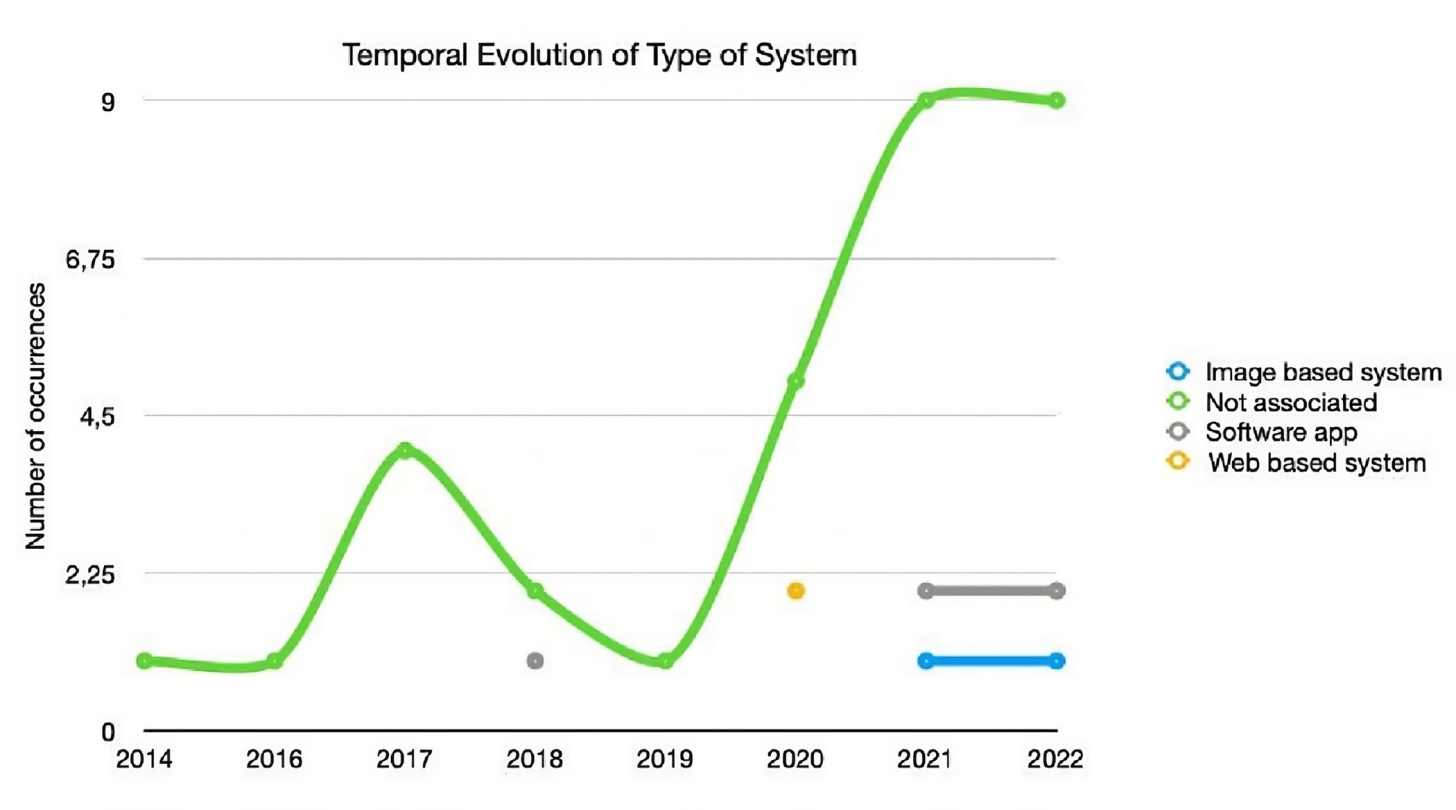
Temporal evolution of type of system.

Finally, the temporal representation of the Type of Systems developed is shown in Fig 7. The existence of studies where the type of systems could be more evident is directly related to the significant representation of Case Studies. Even so, it is possible to identify a slight accentuation of cases where individualised software applications were developed. As of 2021, image-based software applications appear, a trend that should be followed in the coming years.

### 4.5. Trends and autonomy levels

To relate the study with the research objective, it is necessary to assess whether the articles under study reach the deployment phase. Thus, after a detailed analysis of them, it was important to categorize this phase into 4 distinct moments: No/Yes, Partial and Potential implementation. Table 8 presents the number of studies for each of the moments.

**Table 8:** Deployment stage on the reviewed studies.

Study	Outcome	Total	Value
[8, 30, 31, 34, 36, 37, 39, 40, 44, 47–49, 51, 53, 54, 56–59]	No Implementation	19	44.2%
[60, 61, 64]	Partial Implementation	3	7.0%
[24, 28, 29, 32, 33, 35, 38, 41–43, 45, 46, 50, 52, 55, 63, 68]	Potential Implementation	17	39.5%
[62, 65–67]	Yes	4	9.3%

**Fig 8:**
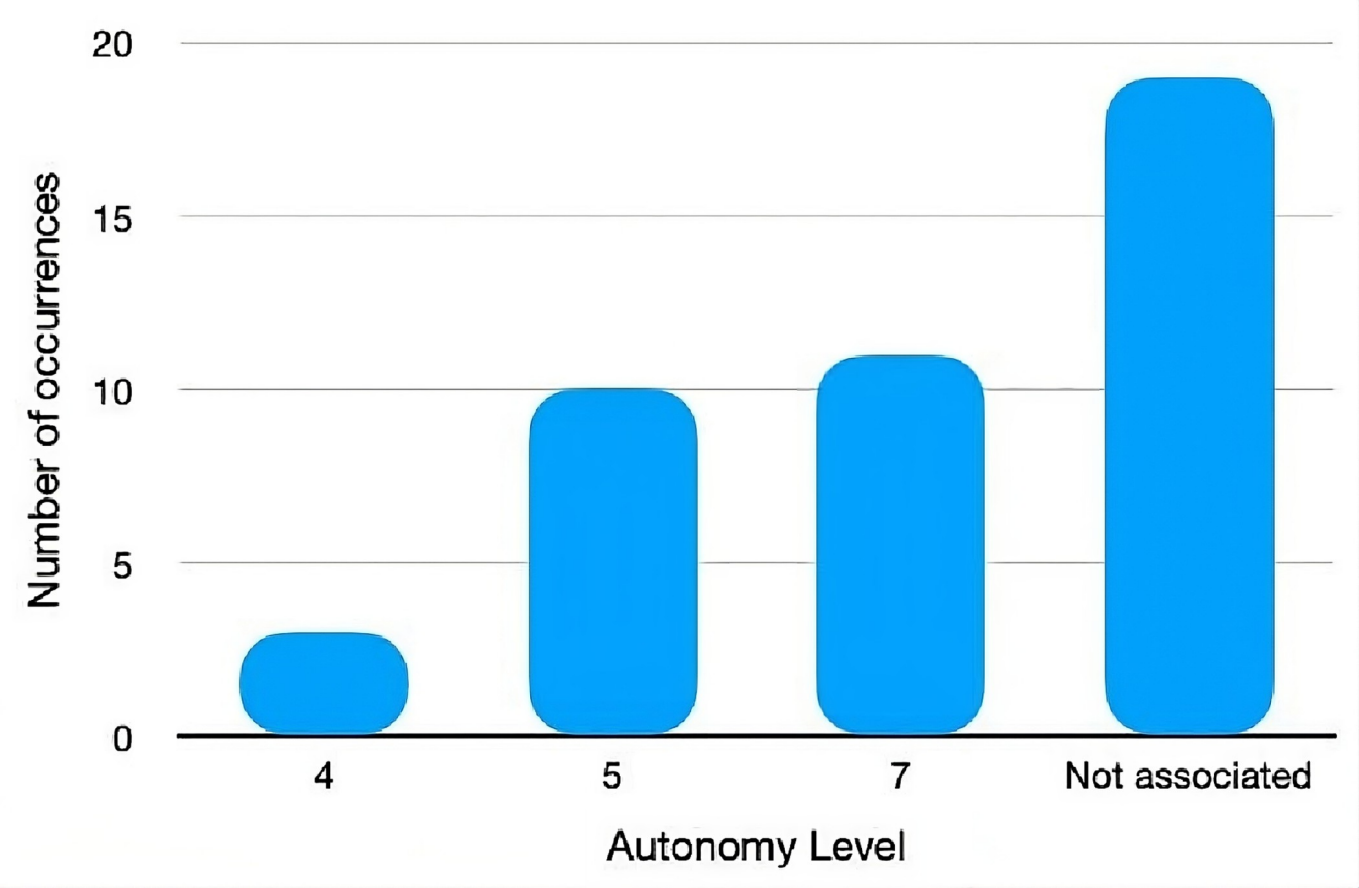
Number of occurrences of autonomy levels.

Fig 8 presents the number of occurrences in the different levels of autonomy, according to Sheridan’s Classification for Levels of Autonomy. Despite the considerable number of articles in this research, it is worth noting the high percentage of studies where it was not possible to draw any conclusion on the attribution of a level of autonomy to the ABI components developed. Even so, it was possible to assign a level of autonomy in 24 studies, of which three were assigned level 4, ten were assigned level 5, and eleven were assigned level 7. Considering the Levels of Autonomy in Table 2, there are no studies in which the developed systems can make decisions automatically without any user interaction (Levels 8–10).

**Fig 9:**
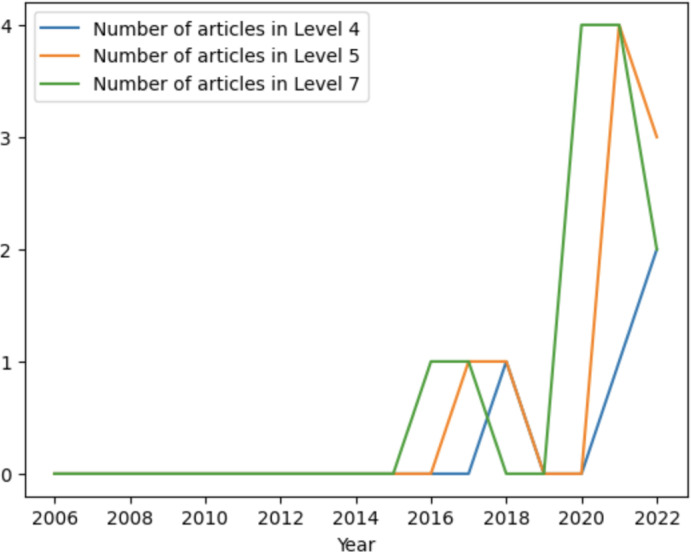
Temporal evolution of autonomy levels.

Fig 9 presents the progression of the three main levels over the years. The data suggests different trends for each level over time. Level 4 and Level 5 seem to remain relatively stable over the last years. On the other hand, Level 7 shows more significant changes, with its most significant peak in 2021. The tendency for the level of autonomy associated with the different components of an ABI system to increase over the last few years is clear.

To conclude, the studies in which some type of implementation or prototype development existed are analysed with the degree of autonomy in each ABI component. Table 9 represent the crossing of Sheridan’s Classification for Levels of Autonomy with the most common characteristics in each group of attributes, based on Table 2, which shows the levels of autonomy considered to be attributed to each type of system. All the studies corresponding to each feature are present in the earlier analyses.

**Table 9:** Weight average on the reviewed studies.

Characteristic		Level 4	Level 5	Level 7
**Study Design**	Case Study	2 (4,7%)	7 (16,3%)	6 (14,0%)
	Prototype Development	1 (2,3%)	3 (7,0%)	5 (11.6%)
**ABI Components**	Predictive	3 (7,0%)	5 (11,6%)	11 (25,6%)
	Prescriptive	0 (0%)	4 (9,3%)	0 (0%)
**Data System Integration**	EHR	0 (0%)	1 (2,3%)	3 (7,0%)
	MHR	0 (0%)	1 (2,3%)	1 (2,3%)
	Specific Database	3 (7,0%)	7 (16,3%)	6 (14,0%)
	Specific Database and EHR	0 (0%)	1 (2,3%)	1 (2,3%)
	Specific Database and MHR	0 (0%)	0 (0%)	0 (0%)
**Featured Algorithms**	Deep Learning	2 (4,7%)	2 (4,7%)	2 (4,7%)
	Logistic Regression	0 (0%)	1 (2,3%)	0 (0%)
	Neural Networks	0 (0%)	0 (0%)	1 (2,3%)
**Type of System**	Image based system	0 (0%)	0 (0%)	2 (4,7%)
	Web based system	2 (4,7%)	0 (0%)	2 (4,7%)
	Software App	0 (0%)	4 (9,3%)	1 (2,3%)

## 5. Discussion

### 5.1 Main findings

The article’s main purpose in question was to develop a systematic review within the scope of Adaptive Business Intelligence (ABI) systems and their different associated components and to study their autonomy in real-life settings. The first research question (RQ1) was to identify the trend of the approaches adopted in developing ABI systems in Health in the analysed studies, as shown in Fig 3. The most pertinent features were chosen in order to categorise the studies: Study Design, Data System Integration, ABI Components, Featured Algorithms and Type of System. The results indicate that the publications at the Study Design level are mostly focused on case studies and prototype creation, with no discernible patterns in the sample collected about their implementation procedures. Concerning integrated data sources, some diversity is verified from the utilisation of specific data sources, EHR and MHR. It’s additionally significant to point out that, in terms of the ABI components, the studies indicate that predictive analytics is more applicable than prescriptive analytics, putting more emphasis on the application of prediction approaches than on potential problem optimisation. Alongside this, the algorithms that stand out the most are Deep Learning, Logistic Regression and Neural Networks. Finally, the research indicates that software applications, web-based, and image-based systems are being developed at the Type of System level. In order to address the second research question (RQ2), a categorisation process was used to determine the degrees of autonomy linked to studies where the different ABI components were actually implemented. As a result, a reference for discussing was created (Table 2), which is based on Sheridan’s Classification for Levels of Autonomy and customised to be used with ABI systems. It consists of ten levels of autonomy that are appropriately categorised to make it simple to frame the system that is being studied. According to this classification, a system is considered completely autonomous when it reaches level 10. However, based on the sample studied, there were few articles where it was possible to associate a level of autonomy due to the absence of a real implementation of the systems developed. Levels 4 and 5 stand out, where the system offers a possible solution for the Healthcare professional, thus presenting these systems with little autonomy, compared to level 7, in which the system already has a higher decision-making capacity, demonstrating significant autonomy, this also being one of the most frequent levels found in the studies analysed. Although there were scientific articles only since 2014, a tendency for the levels of autonomy to increase is evident, with a stabilisation between level 4 and 7 from 2019. In general, highlighting the ABI components, it becomes evident the presence of prescriptive methods only at level 5 of autonomy (9.3%), contrary to the predictive methods, which are present in the various levels under consideration, with a greater weight at level 7 (25.6%). Based on the other characteristics, some similarities can be observed between levels 5 and 7 since both present a greater emphasis on Case Studies (16.3% and 14.0%), integration with specific databases (16.3% and 14.0%) and Deep Learning algorithms (4.7%). In conclusion, level 5 is limited to studies that describe software applications which have been built or may be implemented, whereas level 7 shows an equal distribution of web-based and image-based systems (4.7%).

### 5.2 Limitations and future research

In conducting a systematic review of ABI systems and their relationship with autonomy, some limitations were identified and highlighted in this chapter. Initially, conducting a meta-analysis was not feasible. The limited number of eligible studies in our sample, combined with the diverse types of articles we analysed, made it impossible to conduct a more detailed statistical analysis. A potential limitation of our study could be the exclusion of findings from articles published in languages other than English. This choice may restrict our ability to incorporate insights from non-English literature, and it is important to acknowledge the possibility that relevant information may exist in publications not included in our analysis. Another downside relates to the definition and/or characterization of the ABI components designed, as several studies did not explicitly clarify the methodology used, which could potentially lead to some discrepancies. Likewise, the high number of articles removed from the selected repositories hampered the initial phases of the research process. Not least, it is also common knowledge that the Healthcare has very complex regulations and ethical issues that limit the final deployment of applications. Thus, some difficulties encountered in the implementation of ABI systems and their different developed components may be related to the medical context and its application area, such as: the decision-making capacity; the use of medical/clinical data that hinder the interpretability of results/knowledge; the interoperability between different medical applications and health care information systems; the acceptance of AI as an aid tool and not as a substitute for the role of health professionals. In a future perspective, organisations should work towards overcoming the limitations mentioned above by directing concrete investigations on how the different components of an ABI system should be worked out given its real implementation. One of the perspectives should be data security, which is vital in the ABI approach. This technological approach is considered highly promising for enhancing safety in Healthcare services due to its dynamic and responsive method of analysing data. A notable strength lies in ABI’s capacity to adapt to real-time events enabling the identification and prompt response to potential problems. However, it is imperative to acknowledge potential threats such as data security concerns and ensure the accuracy and reliability of data to prevent erroneous conclusions that could jeopardize patient safety. Seeking a balance between the adaptability and reliability of data sources is essential to fully capitalize on the benefits of ABI. Concerning the deployment stages, it is advisable to initiate the process by carefully considering, in the early stages of development, the targeted level of autonomy that various components should attain (as outlined in Table 2), becoming a crucial requirement for the successful implementation of an ABI system.

## 6. Conclusions

Adaptive Business Intelligence (ABI) systems fundamentally represent a technological structure capable of relating the different types of algorithms used in Artificial Intelligence (AI). To answer the research questions formulated in this work, a systematic review was developed to identify the techniques and approaches, according to levels of autonomy, used in the different components of these systems, in Healthcare, based on 43 studies. The results display clear trends, but the small number of publications makes it impossible to draw meaningful conclusions from several of the features that were evaluated. Even so, it was possible to answer the identified RQs, making it possible to distinguish different levels of autonomy in the face of the various ABI components developed. Additionally, the main trends of the five defined groups were identified: Study Design, Data System Integration, ABI Components, Featured Algorithms and Type of System. It is evident the high number of articles that need to show an approach to the deployment or implementation phases, denoting that there is still much work to be done to make AI a reality in Healthcare. The operational setting presents the most challenges for the implementation of intelligent systems since businesses have not yet fully embraced this approach and have not committed to what should be idealised in the early stages of these studies. Furthermore, based on the gaps identified in this study, new frameworks should be created that make the deployment processes in clinical workflows more straightforward and transparent. In conclusion, this study raises concern about the development of ABI systems since it is notorious for the absence of studies aimed at a structural analysis involving aspects related to autonomy and how their components should be developed, consequently raising awareness among the entities involved about the current state of these systems and how far we are from becoming real use cases. In addition, it allowed us to portray the reality of many systems in the health area, demonstrating that the implementation phase of its components should be idealised in the embryonic stages of the project to anticipate the system’s behaviour in operational clinical environments. This absence may be due to a lack of understanding of what the ideal solution should be, considering the demands of work teams and Healthcare professionals. In conclusion, Sheridan’s classification, which originally appeared more than 20 years ago, remains a useful framework. Sheridan’s categorisation is still used by professionals and academics, providing an essential structure for comparison and study in modern contexts. This article emphasises its applicability by demonstrating how effectively it works across different scenarios. As a reliable point of reference in scientific inquiry, Sheridan’s classification is a useful and timeless framework that continues to influence our understanding of ideas like autonomy and the interactions between technology and people.

## Supporting information

S1 File Information extracted from the review. (DOCX)

S2 File PRISMA checklist. (DOC)

S3 File PRISMA flow diagram of study selection. (DOC)
