# Supplementary material for: Exploring trends and autonomy levels of adaptive business intelligence in healthcare: A systematic review
Source: PLoS One. 2024 May 10;19(5):e0302697. doi: 10.1371/journal.pone.0302697 (PMC11086907; doi:10.1371/journal.pone.0302697)
Supplement: S1 File — (DOCX) [file pone.0302697.s001.docx]

| **Study** | **Study Authors** | **Year** | **Country** | **Data Source** | **Study Design** | **Data System Integration** | **Setting** | **Recipient of Intervention** | **Purpose of Care** | **ABI Components** | **Featured Algorithms** | **Type of System** | **Deployment** | **Autonomy Level** |
| --- | --- | --- | --- | --- | --- | --- | --- | --- | --- | --- | --- | --- | --- | --- |
| [Diagnosis and risk stratification in hypertrophic cardiomyopathy using machine learning wall thickness measurement: a comparison with human test-retest performance](https://www.thelancet.com/journals/landig/article/PIIS2589-7500(20)30267-3/fulltext) | Augusto et al. | 2021 | UK | TLDH | Case study | MHR | Any healthcare setting | Healthcare providers | Automated machine learning algorithm for left ventricular maximum wall thickness (MWT) measurement | Predictive | Linear Regression | Not associated | Potentially implementation | 7 |
| [Development of a clinician-facing prototype for health monitoring using smartwatch data](http://aisel.aisnet.org/acis2022/53) | Bajaj, Ruhi et al. | 2022 | New Zeland | AIS | Prototype development | Specific database | Any healthcare setting | Patients; Healthcare providers | Instrument that detects anomalies and visualizes smartwatch data to improve patient care delivery | Predictive | Clustering | Web based system | Partially implementation | 4 |
| [Predicting Unplanned Hospital Readmissions using Patient Level Data](http://aisel.aisnet.org/hicss-54/hc/big_data_on_healthcare_app/7) | Balan et al. | 2021 | USA | AIS | Case study | Specific database | Hospital | General managers | Predict hospital readmissions | Predictive | Logistic Regression | Not associated | Potentially implementation | 5 |
| Two-stage prediction model for in-hospital mortality of patients with influenza infection | Cheong et al. | 2021 | Taiwan | Pubmed | Case study | Specific database | Not evident | Healthcare providers | Prediction model for mortality of patients with influenza infection | Predictive | Logistic Regression | Not associated | No | Not associated |
| [Development of a treatment selection algorithm for SGLT2 and DPP-4 inhibitor therapies in people with type 2 diabetes: a retrospective cohort study](https://www.thelancet.com/journals/landig/article/PIIS2589-7500(22)00174-1/fulltext) | Dennis et al. | 2022 | UK | TLDH | Case study | MHR | Any healthcare setting | Healthcare providers | Predict the optimal treatment for people with type 2 diabetes | Not evident | Not evident | Not associated | Potentially implementation | 5 |
| Improving healthcare access management by predicting patient no-show behaviour | Ferro et al. | 2020 | UK | DSSJ | Case study | Specific database | Hospital | Healthcare providers | Predict patient no-show behaviour | Predictive | Neural Networks | Not associated | No | Not associated |
| [Translating clinical and patient-reported data to tailored shared decision reports with predictive analytics for knee and hip arthritis.](https://pubmed.ncbi.nlm.nih.gov/32562194/) | Franklin et al. | 2020 | USA | Pubmed | Prototype development | Specific database | Hospital | Healthcare providers; General managers; Patients | Web-based system to collect patient-reported outcomes and medical risk factors and to compare responses to national registry data | Predictive | Multivariate models | Web based system | Partially implementation | 7 |
| [Mathematical Modelling and Cluster Analysis in Healthcare Analytics - The Case of Length of Stay Management](http://aisel.aisnet.org/icis2016/DataScience/Presentations/11) | Gartner and Padman | 2016 | UK | AIS | Case study | EHR | Hospital | Healthcare providers | Identify LOS outliers | Predictive | Clustering | Not associated | Potentially implementation | 7 |
| [Machine learning models for predicting risk of depression in Korean college students: Identifying family and individual factors](https://pubmed.ncbi.nlm.nih.gov/36466485/) | Gil et al. | 2022 | Korea | Pubmed | Case study | Specific database | Not evident | Not evident | Predict college students at risk of depression | Predictive | Logistic Regression | Not associated | No | Not associated |
| [A Decision Support System for Managing Uncertainty in the Delivery of Palliative Care in the Community](http://aisel.aisnet.org/sigdsa2022/14) | Heavin et al. | 2022 | Ireland | AIS | Case study | Specific database | Any healthcare setting | Healthcare providers;  General managers | Assess patient care needs and the stability of a patient’s condition and to help to triage patient | Prescriptive | Bayesian Networks | Software app | Potentially implementation | 5 |
| Personalized predictions of patient outcomes during and after hospitalization using artificial intelligence | Hilton et al. | 2020 | USA | npjDM | Case study | EHR | Any healthcare setting | Healthcare providers | Predict LOS to personalized hospitalizations | Predictive | Gradient Boosting | Not associated | No | Not associated |
| Multisite implementation of a workflow-integrated machine learning system to optimize COVID-19 hospital admission decisions | Hinson et al. | 2022 | USA | npjDM | Prototype development | EHR | Hospital; Any healthcare setting | Healthcare providers; General managers | Estimate short-term risk for clinical deterioration to optimize COVID-19 hospital admission decisions | Predictive | Not evident | Software app | Yes | 7 |
| Development and validation of a deep neural network model to predict postoperative mortality, acute kidney injury, and reintubation using a single feature set | Hofer et al. | 2020 | USA | npjDM | Case study | MHR | Any healthcare setting | Healthcare providers | Predict medical complications during the perioperative period of patients | Predictive | Neural Networks | Not associated | No | Not associated |
| [An autonomous cycle of data analysis tasks for the clinical management of dengue](https://www.sciencedirect.com/science/article/pii/S240584402202134X) | Hoyos, William et al. | 2022 | Spain | PubMed | Case study | Specific database | Any healthcare setting | Healthcare providers | Ability to classify a patient's clinical picture and recommend the best treatment option | Prescriptive | Support Vector Machine; Deep Learning; Genetic Algorithm | Not associated | Potentially implementation | 5 |
| [Deep-learning-based cardiovascular risk stratification using coronary artery calcium scores predicted from retinal photographs](https://www.thelancet.com/journals/landig/article/PIIS2589-7500(21)00043-1/fulltext) | Hyungtaek Rim, Tyler et al. | 2021 | Singapore | TLDH | Case study | Specific database | Any healthcare setting | Healthcare providers | Retinal photographs to predict the presence of CAC | Predictive | Deep Learning | Not associated | Potentially implementation | 4 |
| Machine learning-based in-hospital mortality prediction models for patients with acute coronary syndrome | Ke et al. | 2022 | China | Pubmed | Case study | Specific database; MHR | Hospital; Any healthcare setting | Healthcare providers;  General managers | Prediction models for patients with acute coronary syndrome | Predictive | Gradient Boosting | Not associated | No | Not associated |
| Patient and hospital characteristics predict prolonged emergency department length of stay and in-hospital mortality: a nationwide analysis in Korea | Lee et al. | 2022 | Korea | Pubmed | Case study | Specific database | Hospital | General managers | Predict emergency department length of stay (EDLOS) | Predictive | Logistic Regression | Not associated | No | Not associated |
| Predicting microvascular invasion in hepatocellular carcinoma: a deep learning model validated across hospitals | Liu et al. | 2021 | Taiwan | Pubmed | Prototype development | Specific database | Hospital | Healthcare providers; General managers | Image-based system to predict microvascular invasion in hepatocellular carcinoma | Predictive | Deep Learning | Image based system | Potentially implementation | 7 |
| [WORKLOAD PREDICTION MODEL OF A PRIMARY HEALTH CENTRE](http://aisel.aisnet.org/ecis2017_rp/77) | Lloyd et al. | 2017 | New Zeland | AIS | Case study | Specific database | Any healthcare setting | Healthcare providers | Prediction model for a primary health centre | Predictive | Rule-based algorithm | Not associated | Potentially implementation | Not associated |
| Healthcare in Fraudster's Crosshairs: Designing, Implementing and Evaluating a Machine Learning Approach for Anomaly Detection on Medical Prescription Claim Data | Matschak et al. | 2021 | Germany | AIS | Prototype development | Specific database | Any healthcare setting | General managers | Provides  an applicable method for medical prescription fraud detection | Predictive | Random Forest | Not associated | Partially implementation | 5 |
| A simulation-based evaluation of machine learning models for clinical decision support: application and analysis using hospital readmission | Mišić et al. | 2021 | USA | npjDM | Prototype development | Specific database; EHR | Hospital; Any healthcare setting | General managers | Quantifying the expected patient outcomes and cost savings | Predictive | L1R1; Simulation Models | Software app | Yes | 5 |
| [Development and validation of an artificial neural network algorithm to predict mortality and admission to hospital for heart failure after myocardial infarction: a nationwide population-based study](https://www.thelancet.com/journals/landig/article/PIIS2589-7500(21)00228-4/fulltext) | Mohammad Dennis, John et al. | 2022 | UK | TLDH | Case study | Specific database | Any healthcare setting | Healthcare providers | Identifying patients at a high risk of developing heart failure or death after myocardial infarction | Predictive | Deep Learning | Web based system | Potentially implementation | 4 |
| [Leveraging Time Series Data in Similarity Based Healthcare Predictive Models: The Case of Early ICU Mortality Prediction](http://aisel.aisnet.org/amcis2017/Healthcare/Presentations/11) | Morid et al. | 2017 | USA | AIS | Case study | Specific database | Hospital | Healthcare providers | Early ICU mortality prediction | Predictive | Time-Series | Not associated | Potentially implementation | 7 |
| Predicting scheduled hospital attendance with artificial intelligence | Nelson et al. | 2019 | UK | npjDM | Case study | Specific database | Hospital; Any healthcare setting | Healthcare providers; General managers | Predict hospital attendance | Predictive | Stacking | Not associated | No | Not associated |
| [A Data-analytical System to Predict Therapy Success for Obese Children](https://www.dfki.de/fileadmin/user_upload/import/10116_ICIS_2018_Revison.pdf) | Öksüz et al. | 2018 | Zurich | AIS | Case study | Specific database | Hospital | Healthcare providers | Predict of future BMI changes before conduct a therapy | Predictive | Support Vector Machine | Not associated | No | 4 |
| An integrated deep learning and stochastic optimization approach for resource management in team-based healthcare systems | Olya et al. | 2021 | USA | ESWA | Case study | Specific database | Hospital | Healthcare providers; General managers | Prescriptive approach for resource management | Prescriptive | Deep Learning; Monte Carlo Simulation | Software app | Potentially implementation | 5; 7 |
| [Predicting Fall Risks Vulnerability with Inpatient Data in Acute Care Hospitalization](http://aisel.aisnet.org/amcis2020/data_science_analytics_for_decision_support/data_science_analytics_for_decision_support/1) | Ossai et al. | 2020 | Australia | AIS | Case study | EHR | Any healthcare setting | Healthcare providers; General managers | Predicts fall risks on admission | Predictive | Extra Tree Classifier | Not associated | Potentially implementation | 7 |
| Feature Explanations in Recurrent Neural Networks for Predicting Risk of Mortality in Intensive Care Patients | Pattalung et al. | 2021 | Thailand | Pubmed | Case study | Specific database | Any healthcare setting | Healthcare providers | Predict the risk of mortality in the ICU | Predictive | Deep Learning | Not associated | Potentially implementation | 7 |
| [Data Mining Models for Automatic Problem Identification in Intensive Medicine](https://www.sciencedirect.com/science/article/pii/S1877050922015976) | Quesado et al. | 2022 | Portugal | Pubmed | Case study | Specific database | Any healthcare setting | Healthcare providers | Predict medical problem in intensive medicine | Predictive | Support Vector Machine | Not associated | No | Not associated |
| Predicting adverse outcomes due to diabetes complications with machine learning using administrative health data | Ravaut et al. | 2021 | Canada | npjDM | Case study | Specific database | Any healthcare setting | Healthcare providers | Predict adverse outcomes due to diabetes complications | Predictive | Not evident | Not associated | No | Not associated |
| [Contribution of Different Data Sources to the Prediction of Emergency Department Revisits in a Safety-Net Population](https://web.archive.org/web/20210812103218id_/https:/aisel.aisnet.org/cgi/viewcontent.cgi?article=1261&context=icis2018) | Ryan Vest, Joshua et al. | 2018 | USA | AIS | Case study | EHR | Emergency Department | General managers | Understand the role that EHR and HIE data can play in reducing the probabilities of a patients’ return visit to the emergency department within 30 days | Predictive | Decision Tree | Not associated | No | Not associated |
| Stratifying no-show patients into multiple risk groups via a holistic data analytics-based framework | Simsek et al. | 2020 | USA | DSSJ | Prototype development | Specific database | Hospital | Healthcare providers; General managers | Multiple risk groups idnetification | Predictive | Neural Networks | Web based system | Yes | 7 |
| [Integrated multimodal artificial intelligence framework for healthcare applications](https://www.ncbi.nlm.nih.gov/pmc/articles/PMC9489871/) | Soenksen et al. | 2022 | USA | Pubmed | Prototype development | Specific database; EHR | Any healthcare setting | Healthcare providers | AI Framework and system for healthcare applications | Predictive | Gradient Boosting | Image based system | Yes | 7 |
| Optimizing outpatient appointment system using machine learning algorithms and scheduling rules: A prescriptive analytics framework | Srinivas and Ravindran | 2018 | USA | ESWA | Prototype development | Specific database | Hospital | Healthcare providers; General managers | Outpatient appointment system | Prescriptive | Ensemble Classifier; Simulation Models | Software app | Potentially implementation | 5 |
| Developing and validating a predictive model for future emergency hospital admissions | Stylianou et al. | 2022 | Cyprus | Pubmed | Case study | Specific database | Hospital | General managers | Predictive model for future emergency hospital admissions | Predictive | Logistic Regression | Not associated | No | Not associated |
| [Predicting overdose among individuals prescribed opioids using routinely collected healthcare utilization data.](https://pubmed.ncbi.nlm.nih.gov/33079968/) | Sun et al. | 2020 | USA | PubMed | Case study | Specific database | Any healthcare setting | Healthcare providers | Predict overdose | Predictive | Elastic Net | Not associated | Potentially implementation | 7 |
| [Development of an automated physician review classification system: A novel semi-supervised learning approach](http://aisel.aisnet.org/sigdsa2021/12) | Suresh et al. | 2021 | USA | AIS | Case study | Specific database | Any healthcare setting | Healthcare providers | A classifier to categorize physician reviews as clinical vs. non-clinical texts | Predictive | Neural Networks | Not associated | No | 7 |
| [A Hybrid Mining Approach to Facilitate Health Insurance Decision: Case Study of Non-Traditional Data Mining Applications in Taiwan NHI Databases](http://aisel.aisnet.org/hicss-50/hc/global_health/2) | Tan, Joseph et al. | 2017 | China | AIS | Case study | Specific database | Hospital | General managers | Data mining methods to facilitate claims review processing and provide policy information for insurance decision-making | Predictive | Clustering; Neural Networks | Not associated | No | 2 |
| [Distributed Cognitive Expert Systems in Cancer Data Analytics: A Decision Support System for Oral and Maxillofacial Surgery](http://aisel.aisnet.org/icis2017/IT-and-Healthcare/Presentations/19) | Tofangchi et al. | 2017 | South Korea | AIS | Case study | EHR | Any healthcare setting | Healthcare providers | Predict optimal treatments and communicates its prediction confidence for oral and maxillofacial surgery cases | Predictive | Random Forest | Not associated | Potentially implementation | 5 |
| [Implementation of Predictive Algorithms for the Study of the Endarterectomy LOS](https://pubmed.ncbi.nlm.nih.gov/36290514/) | Trunfio et al. | 2022 | Italy | Pubmed | Case study | Specific database | Hospital | Healthcare providers | Predict Endarterectomy LOS | Predictive | Decision Tree | Not associated | No | Not associated |
| [Clinical validation of an epigenetic assay to predict negative histopathological results in repeat prostate biopsies.](https://pubmed.ncbi.nlm.nih.gov/24747657/) | W. Partin, Alan et al. | 2014 | USA | PubMed | Case study | Specific database | Any healthcare setting | Healthcare providers | Validation the performance of an epigenetic test as an independent predictor of prostate cancer risk to guide decision making for repeat biopsy | Predictive | Logistic Regression | Not associated | No | Not associated |
| An explainable machine learning model for predicting in-hospital amputation rate of patients with diabetic foot ulcer | Xie et al. | 2021 | China | Pubmed | Case study | EHR | Any healthcare setting | Healthcare providers;  General managers | Predict amputation rate of patients with diabetic foot ulcer | Predictive | Gradient Boosting | Not associated | No | Not associated |
| Predicting in-hospital length of stay a two-stage modeling approach to account for highly skewed data | Xu et al. | 2022 | USA | Pubmed | Case study | EHR | Any healthcare setting | Healthcare providers;  General managers | Predict in-hospital LOS | Predictive | Random Forest | Not associated | No | Not associated |
