# Supplementary material for: Exploring trends and autonomy levels of adaptive business intelligence in healthcare: A systematic review
Source: PLoS One. 2024 May 10;19(5):e0302697. doi: 10.1371/journal.pone.0302697 (PMC11086907; doi:10.1371/journal.pone.0302697)
Supplement: S3 File — (DOC) [file pone.0302697.s003.doc]

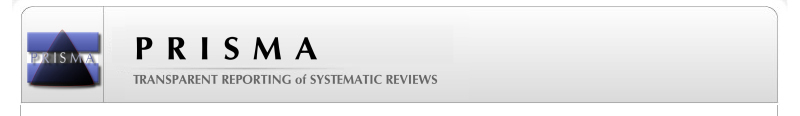
**PRISMA 2009 Flow Diagram**

**Screening**

**Included**

**Eligibility**

**Identification**

PubMed = 2538

DSSJ = 238

Nature = 23

npjDM = 59

ESWA = 250

AISel = 2220

TLDH = 30

(n = 67)

Records
(n = 5328)

Records screened
(n = 4277)

Records excluded (n = 4120):

No open access

No articles

Out of period

No English

Literature Review/Frameworks articles

Full-text articles assessed for eligibility
(n = 157)

Full-text articles excluded
(n = 113):

No Predictive/Prescriptive components from ABI

Not within the scope of the research objective

Studies included in synthesis

(n = 44)

Duplicates recorded
(n = 1051)
